# The Dietetic Value of Pasteurised Milk

**Published:** 1902-05-03

**Authors:** J. Odery Symes

**Affiliations:** Assistant Physician Bristol General Hospital.


### Hospital Clinics and Medical Progress.

### THE DIETETIC VALUE OF PASTEURISED MILK.

By J. Odery Symes, M.D., D.P.H., Assistant Physician Bristol General Hospital.

At the present time there seems to be a general agreement that it is inadvisable to feed young infants on cow's milk in its fresh state, even when properly diluted. The reasons assigned are firstly: that if the cow from which the milk is obtained be suffering from tuberculosis or some modified form of diphtheria or scarlatina, then these diseases may be communicated to the child; and secondly, that during the processes of milking and trade manipulation certain pathogenic and putrefactive organisms are added to the milk, and these when ingested may excite general or gastro-intestinal disorders. The question then arises, To what process should the milk be subjected? Is it desirable that it should be pasteurised, boiled, or sterilised? Sterilised milk is milk which has been heated to a temperature of 105° to 115° centigrade and kept at this temperature for a period varying from 15 to 45 minutes. Although for all practical purposes each exposure to high temperature may be regarded as securing absolute sterilisation, yet it is not uncommon for certain spore-bearing organisms to survive, and consequently the sterilised milk of commerce will not infrequently be found undergoing decomposition.

The absolute sterility of milk is not, however, altogether a thing to be desired. Nuttall and Thierfelder have shown that young animals fed on sterilised food and kept under aseptic conditions do not flourish and put on weight so readily as do similar animals under natural conditions. Bienstock has pointed out that the Bacillus putrificus (an organism which, by setting up putrefactive changes in food, is a frequent exciting cause of diarrhœa) is inhibited by the Bacillus coli and B. lactis ærogenes, so that, by feeding a child suffering from such diarrhœa on sterilised milk we may be actually increasing the evil by excluding the aforementioned organisms, the latter of which (B. lactis ærogenes) is always present in untreated milk. Further, the prolonged exposure to high temperature must destroy the natural ferments in the milk, and these it is supposed bear an important part in the processes of digestion. Apart from these objections based on biological grounds there are others based on the chemical alterations which must necessarily follow in milk which has been subjected to high temperature. These may briefly be summarised as follows:—In the process of sterilisation a scum of coagulable albumen forms, and this, which contains about one-fifth of the total amount of casein, is lost, for the scum is generally removed before using the milk (Winter Blyth). Blackader states that the proteids of sterilised milk are modified and rendered less digestible; the combination of saline ingredients with proteids is more or less broken, and the salts rendered less readily absorbed; the citric salts are altered and precipitated, and the emulsion of the milk is altered and rendered less digestible. Some of these changes are of an unimportant nature and would have little effect upon the nutritive value of the milk, but the effect of the sum total of these alterations is a more serious matter, and there can be no doubt that the nutritional value of the milk suffers materially by the process of sterilisation. More than this, there is at least one constitutional disease, namely infantile scurvy, which may be excited by an exclusive diet of sterilised milk. Cheadle, who first described infantile scurvy, was of opinion that it might arise from exclusive feeding on sterilised milk, although the majority of cases were attributable to farinaceous foods, condensed milk, and humanised milk. Barlow says: "I have had several cases of scurvy in children taking milk sterilised by Soxhlet's process, which takes 40 to 45 minutes." Barton comes to precisely the same conclusion. The arguments which may be brought against the use of sterilised milk do not apply generally to boiled milk, that is milk which has been brought to the boiling point for a minute and then allowed to cool rapidly. A short period of boiling is sufficient to kill all pathogenic organisms which may be present, but does not destroy the spore-bearing saprophytic varieties, which would seem to play some part in the processes of digestion. Milk that has been boiled for a short time does not lose its anti-scorbutic properties, all writers of infantile scurvy agreeing that this disease does not occur amongst children fed solely on boiled milk. The chief objections to boiled milk are that by some children it is not so readily digested, and gives rise to constipation, whilst most children unless brought up from birth upon it object to the taste of the boiled milk. The objections which have been made to the prolonged use of humanised, sterilised, and boiled milks do not apply to the pasteurised product. Pasteurised milk is milk which has been heated for from ten to twenty minutes at a temperature of from 80° to 65° centigrade and which has then been rapidly cooled to 10° centigrade. Such milk possesses several advantages. Its taste is not altered, chemical analysis shows that its albuminous value is not lessened; but although the temperature to which it is raised is sufficient to destroy all pathogenic bacilli, it does not destroy the majority of spore-bearing saprophytic organisms present. Pasteurised milk does not cause scurvy, and this is probably due to the fact that it is not stored for several days like sterilised milk, and consequently does not devolop those minor putrefactive changes which are believed to be the exciting cause of this disease. Young children readily digest pasteurised milk, even when other artificially-prepared products are rejected; they rapidly put on weight, and the constipation which so often results from the use of the boiled article is not noted. Our conclusions briefly summarised may be stated thus:—For the feeding of young children or adults, pasteurised milk is superior to any other preparation, and where this cannot be obtained it is advisable to use milk which has been kept at the boiling point for a few minutes only. The prolonged use of sterilised, humanised, or condensed milks is to be avoided, as these are of lessened nutritive value, and have been shown to be capable of exciting the not uncommon condition of infantile scurvy.

